# At least two Fc Neu5Gc residues of monoclonal antibodies are required for binding to anti-Neu5Gc antibody

**DOI:** 10.1038/srep20029

**Published:** 2016-01-29

**Authors:** Chuanfei Yu, Kai Gao, Lei Zhu, Wenbo Wang, Lan Wang, Feng Zhang, Chunyu Liu, Meng Li, Mark R. Wormald, Pauline M. Rudd, Junzhi Wang

**Affiliations:** 1 National Institutes for Food and Drug Control, Beijing, China; 2 International Joint Cancer Institute, the Second Military Medical University, Shanghai, China; 3 Oxford Glycobiology Institute, Department of Biochemistry, University of Oxford, UK; 4 National Institute for Bioprocessing Reseach and Training, Dublin, ROI; 5 Bioprocessing Technology Institute, A*Star, Singapore

## Abstract

Two non-human glycan epitopes, galactose-α-1,3-galactose (α-gal) and Neu5Gc-α-2-6-galactose (Neu5Gc) have been shown to be antigenic when attached to Fab oligosaccharides of monoclonal antibodies (mAbs) , while α-gal attached to Fc glycans was not. However, the antigenicity of Neu5Gc on the Fc glycans remains unclear in the context that most mAbs carry only Fc glycans. After studying two clinical mAbs carrying significant amounts of Fc Neu5Gc, we show that their binding activity with anti-Neu5Gc antibody resided in a small subset of mAbs carrying two or more Fc Neu5Gc, while mAbs harboring only one Neu5Gc showed no reactivity. Since most Neu5Gc epitopes were distributed singly on the Fc of mAbs, our results suggest that the potential antigenicity of Fc Neu5Gc is low. Our study could be referenced in the process design and optimization of mAb production in murine myeloma cells and in the quality control of mAbs for industries and regulatory authorities.

With annual double-digit growth, mAbs have maintained their ranking as the highest selling class of biologics in recent years^1^. mAbs are frequently manufactured in mammalian expression systems to achieve appropriate glycosylation, and the glycosylation pattern is often critical for the Fc effector functions, providing stability against proteolysis as well as modulating clearance and immunogenicity^2^,^3^.

Rodent cells are the predominant choices for the production of mAbs, particularly Chinese Hamster Ovary cells (CHO cells) and two murine myeloma cells (SP2/0 and NS0 cells)^4^. The rodent cells have the genetic makeup necessary to add two non-human glycan structures, galactose-α-1,3-galactose (α-gal) and N-glycolyneuramic acid (Neu5Gc), to the glycan terminus of mAbs^5^. CHO cells express mAbs with an undetectable or trace level of the unnegligible α-Gal^6^ and Neu5Gc^7^, while murine myeloma cells including SP2/0 and NS0 usually express much higher level of the two glycan structures^8^,^9^. Humans are genetically deficient in the orthologous gene, *N*-acetyllactosaminide-3-α-galactosyltransferase-1, which is responsible for the biosynthesis of terminal α-Gal^10^. In addition, humans have an irreversibly mutated gene, CMP-*N*-acetylneuraminic acid hydroxylase (*CMAH*), which is responsible for CMP-Neu5Gc production from CMP-N-acetylneuraminic acid (CMP-Neu5Ac)^11^. Pre-existing anti-α-gal IgE mediated anaphylactic responses^12^ and pre-existing anti-Neu5Gc antibody-mediated immune reactions^9^ have also been reported.

Sialic acids are a group of derivatives of a negatively charged acidic sugar, and the family can be represented by Neu5Ac, Neu5Gc, and deaminoneuraminic acid (KDN)^13^. Sialic acids can influence the biological and physical properties of biopharmaceutical proteins and living cells, including the ability to mask epitopes on underlying glycan chains and to repulse negatively charged moieties^14^. Compared to other species except chicken^15^, the lack of Neu5Gc expression has various pathophysiological implications^16^,^17^,^18^. Exclusive expression of Neu5Ac in ferrets, as in humans, contributes to the susceptibility of the animals to human-adapted IAV strains^19^. However, the possible effect of Neu5Gc on biotherapeutics is poorly documented.

Most mAbs have only two N-linked oligosaccharides, each of which is attached to asparagine at position approximately 297 on each of the two heavy chains that make up the Fc region^20^, however Cetuximab, a chimeric antibody directed to the EGF receptor, produced in SP2/0 cells, has both two Fc glycans and two Fab glycans^21^. Antigenicity has been documented for both terminal α-Gal^12^ and Neu5Gc^9^ on the N-glycans in the Cetuximab Fab, while terminal α-Gal on the Fc glycan of other mAbs expressed from murine cells was not antigenic^8^, but whether terminal Neu5Gc on the Fc glycan of mAbs is antigenic or not remains unclear.

About 40% percent of currently marketed mAbs in the US and the EU are produced in murine myeloma cells^4^, and they are likely to carry varying amounts of Neu5Gc. All humans have circulating anti-Neu5Gc antibodies, sometimes at high levels^22^, and mAbs are often administered at high quantities, usually scores to hundreds of milligrams per dose, over long periods of time. In the development of mAb biosimilar, Fc oligosaccharides terminating in Neu5Gc were documented as a potential immunogen^23^. The above contexts suggest that it is critical to elucidate whether the Fc Neu5Gc is antigenic or not.

## Results

### Only a small subset of Fc Neu5Gc of mAbs binds to the anti-Neu5Gc antibody

We first quantified the two kinds of sialic acids, Neu5Gc and Neu5Ac, in eight mAbs expressed in SP2/0 cells and eight mAbs expressed in NS0 cells, using fluorescent detection of DMB-labeled acid-released sialic acids from the mAbs. As shown in Supplementary Table 1, the sixteen mAbs showed highly varying levels of Neu5Gc. For further study we selected two marketed mAbs, Ustekinumab and Ramucirumab expressed in SP2/0 and NS0 cells respectively which contained the highest levels of Fc Neu5Gc. Ustekinumab^24^, an IgG1/κantibody targeting the common p40 subunit of IL-12 and IL-23, carries 0.524 mol Neu5Gc and only 0.004 mol Neu5Ac per mol of antibody, and Ramucirumab^25^, an IgG1/κantibody targeting VEGFR2, contains 0.109 mol Neu5Gc and only 0.002 mol Neu5Ac per mol of antibody. We discounted the influence of Neu5Ac in our analyses since it was only detected in trace amounts.

**Figure 1:**
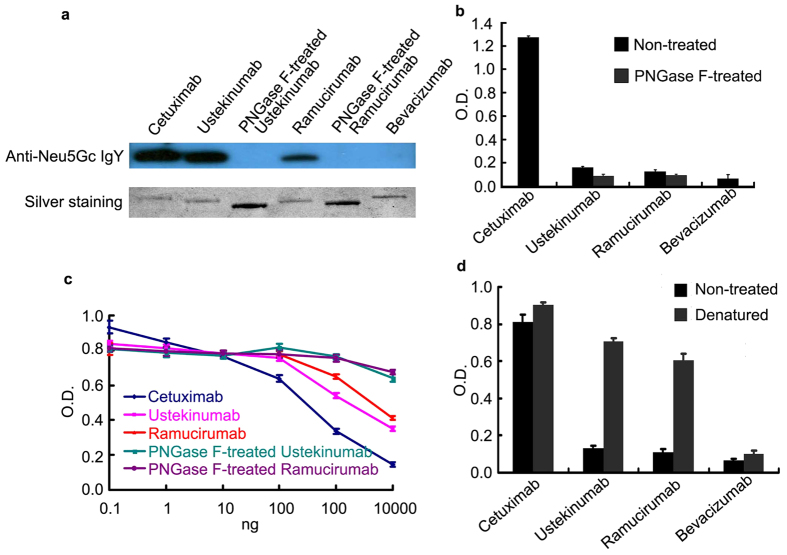
Western-Blot and ELISA Detection of Neu5Gc on various mAbs by Anti-Neu5Gc IgY. (**a**) The mAbs were separated by SDS-PAGE, silver stained to ensure the similar loading amounts of the samples (the two PNGase F-treated samples were loaded more to further ensure the non-reactivity of the samples to the anti-Neu5Gc antibody), and blotted with the anti-Neu5Gc antibody to check the corresponding binding reactivity with the anti-Neu5Gc antibody, (**b**) The samples were biotinylated, captured by streptavidin-coated plates and subjected to ELISA analysis to check their binding reactivity to the anti-Neu5Gc antibody. In an additional ELISA (**c**), the samples competed with the Cetuximab coated on the plates to bind to the anti-Neu5Gc IgY, and the inhibitory binding reactivity was assayed. In another ELISA (**d**), the binding capacity to the anti-Neu5Gc antibody was compared between untreated and guanidine-denatured biotinylated mAbs captured by streptavidin-coated plates.

Western blot analysis using an affinity-purified polyclonal chicken anti-Neu5Gc antibody preparation that is highly mono-specific for Neu5Gc^9^ (Fig. 1a), showed anti-Neu5Gc IgY reactivity on the Cetuximab, Ustekinumab and Ramucirumab heavy chains, but not on PNGase F^26^ treated Ustekinumab, Ramucirumab and Bevacizumab (expressed in CHO cells) heavy chains. These data demonstrate the specificity of the anti-Neu5Gc antibody for the glycans. In our assays, PNGase F removed nearly 100% of the N-glycan on Ustekinumab and Ramucirumab heavy chains as shown by sodium dodecyl sulfate–polyacrylamide gel electrophoresis (CE-SDS) (Supplementary Fig. 1a,b). In the ELISA assay, to avoid the denaturing effect of the highly hydrophobic coating surface of the 96-well plates, we captured the biotin-labeled antibodies with strepavidin coated plates, and evaluated the binding ability of the antibodies with anti-Neu5Gc IgY. Neu5Gc on both Ustekinumab and Ramucirumab could bind to the anti-Neu5Gc antibody as shown by the comparison with their PNGase F- treated counterparts (Fig. 1b). Binding of the anti-Neu5Gc antibody to the coated Cetuximab was inhibited in a dose-dependent manner by both Ustekinumab and Ramucirumab but not their PNGase F-treated counterpart, which also demonstrated the binding specificity of Neu5Gc on the mAbs to the anti-Neu5Gc antibody (Fig. 1c). Notably, binding of Ustekinumab and Ramucirumab to the anti-Neu5Gc antibody was minimal compared to Cetuximab (Fig. 1b), which suggested that most of the Fc Neu5Gc of the two mAbs might be shielded from binding to the anti-Neu5Gc IgY. However, when denatured, the binding reactivity of Neu5Gc on the two mAbs was significantly increased (Fig. 1d), probably due to the exposure of the hidden Neu5GC in the Fc region, consistent with our proposed shielding effect.

### Only the SNA-bound fractions of mAbs bind with the anti-Neu5Gc antibody

IVIG (IntraVenous ImmunoGlobulin) Fc fractionated with Sambucus nigra agglutinin (SNA), a sialic acid binding lectin, was the primary anti-inflammatory component of IVIG^27^,^28^, and only Fc regions containing two or more sialic acids were capable of binding SNA due to the spatial constraint of C_H_2 domain and sialic acid exposure on the surface of the antibody^29^,^30^. The same mechanism might explain the limited binding of Fc Neu5Gc to the anti-Neu5Gc antibody.

**Figure 2:**
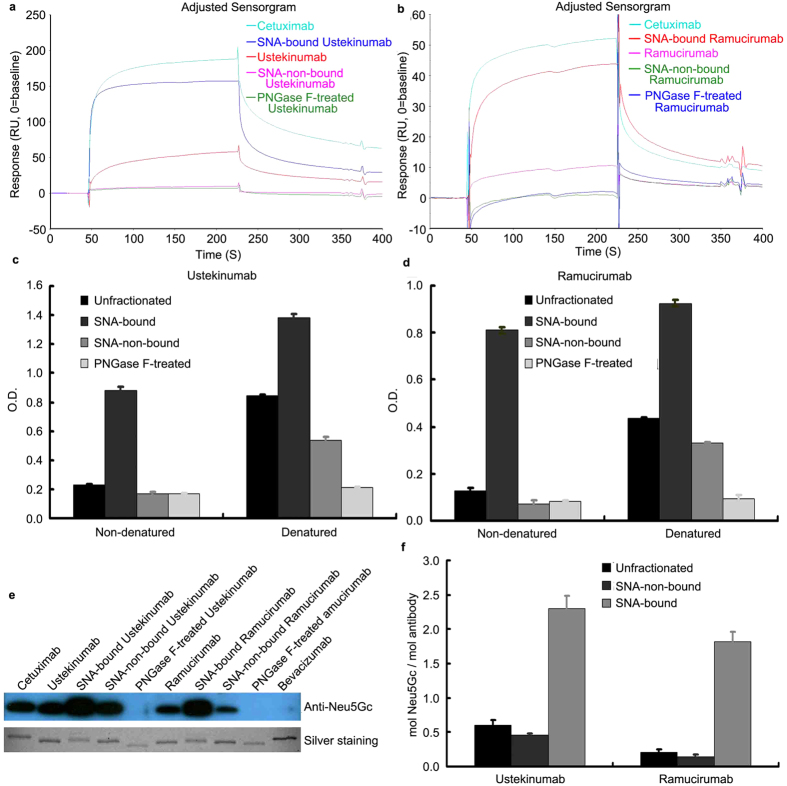
The Binding Reactivity to Anti-Neu5Gc antibody of unfractionated, SNA-non-bound and SNA-bound Ustekinumab and Ramucirumab with BIAcore and ELISA Analysis. Ustekinumab and Ramucirumab were fractionated with SNA, a sialic acid binding lectin. Fractions were assigned as SNA-bound and SNA-non-bound fractions. (**a,b**) Anti-Neu5Gc IgY was immobilized to the CM5 microchip, and the binding kinetics of the mAb samples to the anti-Neu5Gc IgY were assayed by flowing through the chip, in which Cetuximab was the positive control and PNGase F-treated Ustekinumab and Ramucirumab were the baseline negative controls. (**c,d**) Untreated and guanidine-denatured samples were biotinylated, captured by streptavidin-coated plates and subjected to ELISA analysis with the anti-Neu5Gc IgY to check the binding reactivity. (**e**) The samples were separated by SDS-PAGE, silver stained to ensure the similar loading amounts of the samples, and blotted with anti-Neu5Gc IgY to check the corresponding reactivity. (**f**) DMB-labeled acid-released Neu5Gc from the mAb samples were subjected to C18 reverse phase separation and subsequent fluorescent detection and quantification.

As shown by BIAcore analysis in Fig. 2a,b, SNA-bound Ustekinumab and Ramucirumab fractions showed high binding capacity for the anti-Neu5Gc IgY immobilized to the chip, comparable to Cetuximab, while both SNA-non-bound fractions showed similar binding kinetics to their PNGase F-treated counterparts. This suggests that SNA and the anti-Neu5Gc antibody had similar binding patterns to the Fc Neu5Gc of the two mAbs and that there was no binding reactivity to anti-Neu5Gc antibody for either SNA-non-bound fraction. ELISA assays (Fig. 2c,d) also showed similar data to the BIAcore analysis under non-denaturing conditions, however, when denatured with guanidine, both SNA-non-bound fractions showed similar binding activity to the anti-Neu5Gc IgY as their unfractionated counterparts. These data suggest that the antibodies contain similar amounts of Fc Neu5Gc although shielded from binding to the anti-Neu5Gc antibody before denaturation. Western blot analysis (Fig. 2e) and sialic acid quantification assay with the DMB-labeling method (Fig. 2f) indicated that both SNA-non-bound fractions had comparable amounts of Fc Neu5Gc as their unfractionated counterparts, explaining the similar binding reactivity to the Neu5Gc antibody of the two denatured components.

### The subset of mAbs binding with the anti-Neu5Gc antibody and SNA carry no less than two Fc Neu5Gc

**Figure 3:**
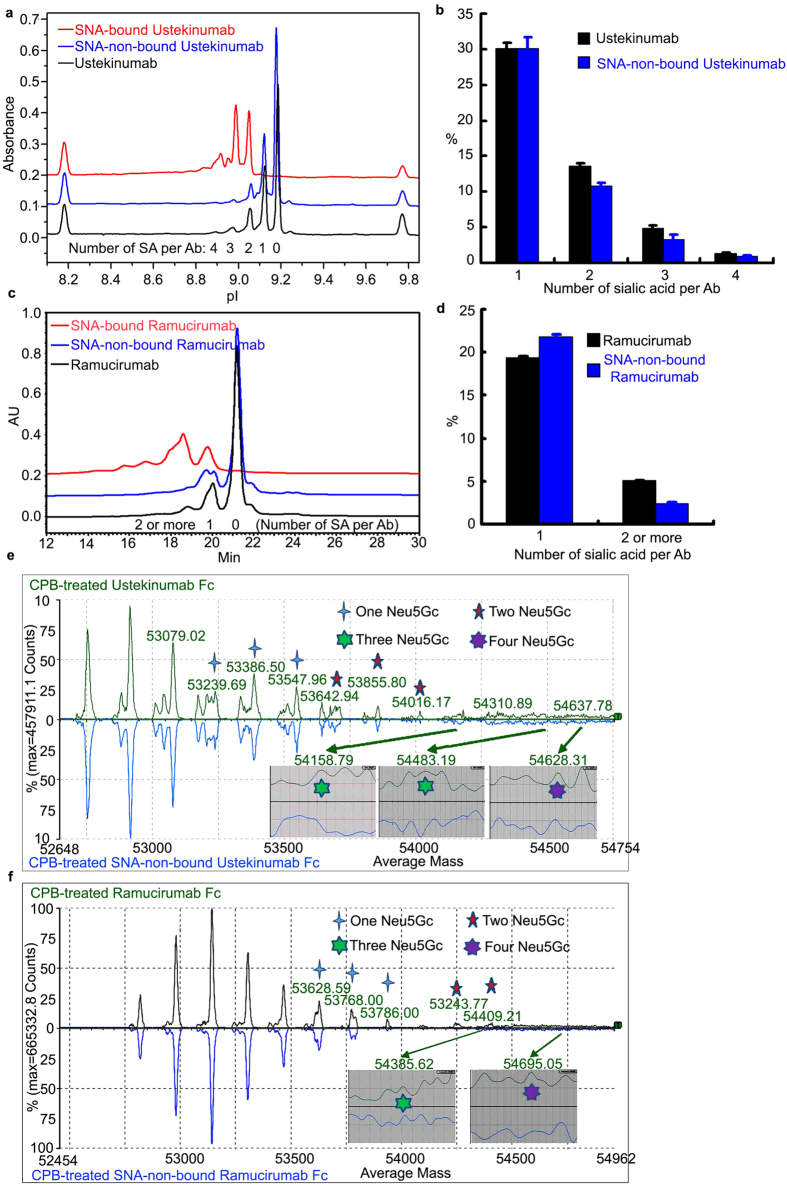
Comparison of Fc Neu5Gc distribution on unfractionated and SNA-non-bound Ustekinumab and Ramucirumab by icIEF, IEC-HPLC and mass spectrometry. Unfractionated, SNA-non-bound and SNA-bound Ustekinumab were treated with CPB to remove c-terminal lysine, hence lysine-induced basic heterogeneity, and the three samples were subjected to icIEF (**a**) and IEC-HPLC (**c**) analysis. The peaks from left of the main peak (no sialic acid) of unfractionated Ustekinumab contained antibodies carrying 1–4 sialic acids in turn (**a**), and their percentage was statistically compared between CPB-treated unfractionated and SNA-non-bound Ustekinumab (**b**). The peaks from left of the main peak (no sialic acid) of unfractionated Ramucirumab were antibodies carrying 1 and 2 or more sialic acids (**c**), and their percentage was statistically compared between CPB-treated unfractionated and SNA-non-bound Ramucirumab (**d**). (**e,f**) Unfractionated and SNA-non-bound antibodies were fragmented with papain into Fab and Fc. They were consecutively trimmed of c-terminal lysine to reduce their heterogeneity, and then subjected to C4 reversed phase separation and mass spectrometric analysis. The Fc spectrum was deconvoluted and peaks with relatively high intensity containing 1 to 4 Neu5Gc were assigned according to the primary structures and combination of two glycoforms shown in Supplementary Figs 4a and 5a.

Due to the similar amounts of Fc Neu5Gc in the unfractionated and SNA-non-bound fractions of Ustekinumab and Ramucirumab, we considered that the Neu5Gc distribution on the Fc of the two mAbs might underlie the differential binding activity to anti-Neu5Gc IgY between the two components. Incomplete c-terminal lysine removal^31^ and differential Fc N-glycan sialylation are the two main sources of mAb charge heterogeneity. These were resolved by imaged capillary isoelectric focusing (icIEF) and ion exchange chromatography-high-performance liquid chromatography (IEC-HPLC)^32^. Carboxypeptidase B (CPB) abolished c-terminal lysine-induced basic variants^31^. PNGase F treatment following CPB digestion^26^ eliminated N-glycan terminal sialylation-induced acidic variants of Ustekinumab (Supplementary Fig. 2a). We could then infer that the peaks eluting before the main peak of CPB-treated Ustekinumab were antibodies containing from one to four sialic acids, although the first acid peak contained a small percentage of other forms of acidic variants. Consecutive CPB and PNGase F treatments of SNA-bound and -non-bound fractions showed similar results (Supplementary Fig. 2b,c). We also noted that PNGase F treatments removed Fc glycans by cleaving the bond between asparagine and the first N-acetyl glucosamine residue at the reducing end of the N-glycan^26^ (Supplementary Fig. 1a,c,d), adding two negative charges to the mAbs due to the conversion of asparagines to aspartic acids (Supplementary Fig. 2a–c). By comparing icIEF electrophoregrams of the CPB-treated unfractionated and SNA-non-bound Ustekinumab (Fig. 3a), we determined the relative percentage of antibody with 0–4 Fc Neu5Gc residues, and then compared the changes in their distribution patterns (Fig. 3b). The percentage of antibody with one sialic acid remained largely unchanged, while those with two-four sialic acids decreased.

Similar to icIEF analysis, carboxypeptidase B (CPB) treated Ramucirumab abolished c-terminal lysine-induced basic variants^31^, and consecutive EndoF2 treatment after CPB digestion trimmed off the N-glycans^26^ (Supplementary Fig. 3a). The EndoF2 treatment simultaneously eliminated N-glycan terminal sialylation-induced acidic variants although a relatively small percentage of acidic variants remained as showed by IEC-HPLC (Supplemetary Fig. 4b). From this we inferred that the peaks to the left of the main peak of CPB-treated Ramucirumab were antibodies containing glycans with one to four sialic acids. We used EndoF2 instead of PNGase F because EndoF2 cleaves the glucosidic bond between the first and second N-acetyl glucosamine residues at the reducing end of N-glycan^26^. This digestion does not significantly influence the elution time of the main peak since it does not result in any dramatic change of surface charge of the mAb, and in our experience, it facilitates the identification of acidic variants induced by sialic acids. Similarly, by comparing IEC-HPLC chromatograms of CPB-treated unfractionated and -SNA-non-bound Ramucirumab (Fig. 3c), we could check the sialic acid distribution pattern change on the Fc (Fig. 3d). Since the peaks including three or four sialic acids were too low to be integrated, we integrated the peaks including 2–4 sialic acids together, which showed similar results as icIEF analysis.

To further verify the conclusion drawn from the icIEF and IEC-HPLC analysis, we studied the Neu5Gc distribution on antibody Fc with mass spectrometry. First, we labeled the PNGase F-released N-glycans from Ustekinumab and Ramucirumab with fluorescent RapiFluor-MS™ Reagent (Waters), and subjected them to HILIC-UPLC separation using fluorescence detection (Supplementary Figs 4a and 5a) followed by MS detection for qualification (Data not shown), which showed that SNA-bound fractions of the two mAbs carry high level of sialylated glycans. We should also note the sialylation level was less than that detected with the DMB-labeling method, since glycan labeling conditions might underrepresent the proportion on sialylated glycans^33^.

To reduce the heterogeneity of the Ustekumumab and Ramucirumab in order to simplify the analysis, we fragmented both antibodies into Fab and Fc with papain digestion, trimmed their C-terminal lysines with CPB treatment, and subjected them to UPLC separation using a C4 reversed-phase column coupled with mass spectrometry analysis. The Fc peaks in the mass spectrogram of the two antibodies were deconvoluted and assigned one by one in accordance with the combination of two of above-mentioned known glycoforms (Fig. 3e,f). Due to the complexity of each glycoform containing 0–2 Neu5Gc, we could not assign all of the peaks, but still we could see that the percentage of SNA-non-bound Ustekinumab and Ramucirumab carrying 2–4 Neu5Gc was significantly reduced. The antibodies carrying one Neu5Gc remained largely unchanged compared with their unfractionated counterparts, which is consistent with the results of the icIEF analysis for Ustekunummab and IEC-HPLC analysis for Ramucirumab.

Furthermore, higher levels of mono-sialylated N-glycans in the SNA-bound than that in the SNA-non-bound mAbs (Supplementary Figs 4 and 5) suggest that the anti-Neu5Gc antibody might bind two monosialylated N-glycans located at each of the two Fc regions of the same mAb molecule. However, given that the disialylated N-glycans are at moderate levels in both SNA-bound mAbs and very low level in SNA-non-bound Ustekinumab but not detectable in SNA-non-bound Ramucirumab (Supplementary Figs 4 and 5), it is also possible that the anti-Neu5Gc antibody could only bind to the disialylated N-glycans located at only one Fc region of the mAb. Further studies are needed to address the question whether the Neu5Gc antibody could bind two monosialylated N-glycans at two Fc regions of the same mAb or only one disialylated at one Fc region of the mAb. Nevertheless, regardless of their distribution, at least two Fc Neu5Gc residues of mAbs are required for their binding to the anti-Neu5Gc antibody.

## Discussion

Although the rapid development of mAbs helps to treat various diseases, their infusion-related side effects and immunogenicity are major concerns^34^. The incidence and severity of an immune reaction varies depending on the interplay between the infused mAb and an individual patient. Formation of antigen-antibody immune complexes could reduce the antibody half-life by being cleared by Fc and mannose receptors on phagocytes in the liver or spleen^35^ and/or induce classical complement activation with resultant inflammation and tissue damage^36^,^37^. Moreover, their deposition in various tissues might induce vascular inflammation, infusion reactions, glomerulopathies, and other potentially adverse effects^36^,^38^. It was documented that the consumption of Neu5Gc-containing red meat might induce the antigen-antibody immune complex related chronic inflammation and tumor progression^17^. The high titer of anti-Neu5Gc IgG is often accompanied by a high tire of high-affinity anti-Neu5Gc IgM in patients with hypothyroidism, which suggests that the immune complex-related immunologic reactions are induced by Neu5Gc in these patients^18^. It should also be noted that Neu5Gc-containing N-Glycolyl GM3 cancer vaccine^39^ and anti- N-Glycolyl GM3 mAb^40^ have been developed to treat cancers due to the higher expression level of Neu5Gc in malignant cells compared to the normal cells. The possible immune complex formation between mAb carrying Fc Neu5Gc and pre-existing anti-Neu5Gc antibody might induce significant side effects, especially when considering that murine myeloma cells express up to 0.5 mol Neu5Gc per mol antibody (Supplementary Table 1). SP2/0 and NS0 cells express much more Fc Neu5Gc glycans than CHO cells. In order to explore the Fc Neu5Gc binding reactivity to its corresponding antibody, mAbs containing higher levels of Neu5Gc expressed from SP2/0 and NS0 cells, such as Ustekinumab and Ramucirumab, were studied.

In our study, SNA-bound fractions of both Ustekinumab and Ramucirumab showed similar significant binding to the anti-Neu5Gc IgY that was comparable to Cetuximab. IcIEF and IEC-HPLC analysis revealed that these binding fractions bore at least two Fc Neu5Gc residues. On the contrary, SNA-non-bound fractions of the two mAbs showed no binding to the anti-Neu5Gc IgY, while icIEF, IEC-HPLC and mass spectrometric analysis revealed that the Fc Neu5Gc distribution patterns between non-fractionated mAbs and SNA-non-bound fractions were different, although they had a similar level of Neu5Gc as showed by Western blot and HPLC quantification assays. The different pattern distributions indicated that antibodies bearing only one Fc Neu5Gc could not bind the anti-Neu5Gc antibody, while a subset of antibodies bearing two-four Fc Neu5Gc could.

Dalziel *et al.* showed that SNA displayed binding with the Fc sialic acid of the antibody in its denatured but not native form^41^. Stadlmann *et al.* further proved that SNA could only bind with antibody bearing two or more Fc sialic acids, indicating that the antibody in Dalziel *et al.*’s study mainly carried only one Fc sialic acid. This may be because CH2 domains allow accommodation of one sialic acid in a conformation in which this residue is hardly accessible to recognition by SNA or lectin-like proteins^29^. From our study, the anti-Neu5Gc seems to be lectin-like since SNA and the anti-Neu5Gc antibody could bind with the same subset of the two mAbs. Our study demonstrated that only a proportion of mAbs bearing two or more Neu5Gc showed binding capacity with SNA and the anti-Neu5Gc antibody, and further studies are entailed to unravel the mechanism.

Considering the spatial constraints in the Fc galactose pocket^42^, sialylation is not easy, especially in case of di, tri or tetra-sialylation^30^ on the Fc glycans. Statistically, 10% of the monosialylated Fc glycoform would contribute to 1% of intact whole disialylated antibody, and as in our study, only less than half of the disialylated antibody could bind to the Neu5Gc antibody. Half-life shortening and/or immune complex related side effects induced by immune complexes formed between pre-existing anti-Neu5Gc antibody and mAbs carrying Fc Neu5Gc might be not a big concern unless a high level of Fc Neu5Gc is present on mAbs expressed from murine myeloma cells. In practice, most mAbs carry less than 0.1 mol Fc Neu5Gc per antibody, as demonstrated by our Neu5Gc quantification assay for the 16 mAbs expressed from murine myeloma cells (Supplementary Table 1). In the case of mAbs expressed from CHO cells, very little sialylation is present ( < 2%)^4^, which resulted in minimal or undetectable levels of Fc Neu5Gc, and the corresponding antigenicity might therefore be ignored. Increase of Neu5Gc content in recombinant human erythropoietin (EPO) might enhance its immunogenicity in a chicken model^43^, and the immunogenicity of Neu5Gc on the N-glycans was also reported in a Cmah null mouse model^9^. Murine myeloma cells are seldom employed in the production of biotherapeutics except mAbs, hence the lower level of Neu5Gc content^9^, which might be the reason why no Neu5Gc related adverse reactions were reported in the clinic. However, the adverse effects induced by mAb with its Fc carrying Neu5Gc were not documented in the clinic even in the laboratory context, probably due to the low antigenicity of Neu5Gc residue partially protected by the CH2 domains. Compared to the hidden Fc Neu5Gc, the solvent-accessible Fab Neu5Gc of Cetuximab could bind with the anti-Neu5Gc antibody more strongly in the native form, (Fig. 1), further explaining the lack of documentation of Fc Neu5Gc antigenicity.

Highly sialylated (α-2,6 lingkage) Fc of IVIG are proved the major player in suppressing inflammation^27^,^28^, and this opens the door to recombinant antibodies with highly sialylated Fc to replace IVIG in treating autoimmune diseases. CHO cells produce mAbs carrying sialic acids only with α-2,3 linkage, since the cells only express α-2,3 sialyltransferase and not α-2,6 sialyltransferase^44^. Furthermore, CHO cell derived sialic acids are mainly in the Neu5Ac form rather than Neu5Gc, and optimization of culture conditions can further decrease the level of Neu5Gc. Using an antisense-RNA strategy, activity reduction of CMP-Neu5Ac hydroxylase, the key enzyme in converting Neu5Ac to Neu5Gc, could markedly decrease the level of Neu5Gc in engineered CHO cells^7^,^45^. Murine myeloma cells, including SP2/0^46^ and NS0^47^ cells, seem to produce mAbs with only α-2,6 lingkage^48^. Furthermore, murine myeloma cell derived sialic acids are mainly in the Neu5Gc form as documented^49^ and according to our data (Supplementary Table 1), while optimization of culture conditions could also further decrease the level of Neu5Gc^9^. Considering the linkage of sialic acids between different expression systems, murine myeloma cells are likely to be the best system for producing mAbs with highly sialylated Fc, while our study strongly indicates the high antigenicity for this kind of mAb, due to the high levels of Neu5Gc. Also it should be noted that engineered CHO cells provide an option to produce highly α2,6-sialylated IgG, with the structure similar to that of highly sialylated IVIG^50^.

Overall, we concluded that only a minor fraction of Neu5Gc on the mAbs carrying two or more Fc Neu5Gc could bind to anti-Neu5Gc antibody. This provides a foundation for the process design and optimization of mAb production in murine myeloma cells, as well as the quality control and the corresponding biosimilar development of mAbs.

## Methods

### Sialic acid quantification with DMB labeling

Sialic acid quantification was performed according to the manufacturer’s instruction (Takara). Briefly, sialic acids were released from 150 μg of each mAb in 50 μl of 0.05 N HCl at 80 °C for 1 hour, 200 μl of mixed solution (mixture of DMB solution, coupling solution and H_2_O as the volume ratio of 1:5:4) were added to the sample, and incubated at 50 °C for 2.5 hours under protection from light, and then cooled on ice for 5 min to terminate the reaction. The labeled samples were resolved on a reversed phase Agilent 300SB-C18 column (4.6-mm internal diameter, 25 cm, 3.5 μm) and 40-min isocratic elution in 9% acetonitrile and 7% methanol/water at a flow rate of 1.0 ml/min (Waters e2695 HPLC). Serially diluted Neu5Gc and Neu5Ac were also labeled as described above, and standard curve was drawn to calculate the mol of Neu5Gc and Neu5Ac per mol mAb.

### PNGase F treatment of mAbs

One milligram of each mAb referred to in the paper was treated with 5,000 U of N-Glycosidase F (also known as PNGase F, NEW ENGLAND Biolabs) in 50 mM ammonium bicarbonate, pH 8.0, at 37˚C for 24 h. The N-glycan removal efficiency was performed with reduced CE-SDS and the treated samples were used for Western Blots, ELISA, or BIAcore and icIEF analysis, as described below.

### EndoF2 treatment of mAbs

One milligram of each mAb was treated with 25 μg of endoglycosidase F2 (also known as EndoF2, gift of Prof. Yajun Guo from the Second Military Medical University, China) in 40 mM ammonium formate, pH 4.5, at 37 °C for 24 h. The N-glycan removal efficiency was performed with reduced CE-SDS, and the treated samples were used for IEC-HPLC analysis, as described below.

### CPB treatment of mAbs

The antibody samples were treated with carboxypeptidase B (also known as CPB, NEW ENGLAND Biolabs) with the weight ratio of 100:1 in the original buffer of the samples at 37˚C for 30min, and the treated samples were used for icIEF, IEC-HPLC and mass spectrometry analysis.

### CE-SDS analysis

To confirm the efficiency of PNGase F or EndoF2 treatment of the antibody samples, capillary electrophoresis-sodium dodecyl sulfate polymer-filled capillary gel electrophoresis (also known as CE-SDS) analyses were performed. 1 mg/mL of each mAb sample in sample buffer (Beckman Coulter) containing 5 μl 2-ME (sigma) with a final volume of 100 μl was heated at 70 °C for 10 min, and then the solution mixture was subjected to CE-SDS analysis with a Beckman PA800 CE system equipped with UV diode-array detector (220 nm wavelength) and a bare-fused silica capillary with L_D_ = 20 cm, L_T_ = 30.2 cm, an inner diameter of 50 μm and outer diameter of 375 μm.

### Lectin affinity column purification

Agarose-bound SNA lectin (Vector Laboratories) was used for the isolation of SNA-bound and SNA-non-bound fractions of the mAbs. Forty milligrams of each mAb in 4 ml of phosphate buffer solution (PBS, pH 7.4) were applied to 4ml of SNA column resin. After incubation at room temperature for 1 h, the flow-through fraction was collected, and the SNA column was washed with 16 ml of PBS. The mAbs bound to the SNA column were eluted and collected after incubation in 4 ml of 0.5 M lactose in PBS for 30 min and the same amount of 0.5 M lactose in 0.2 M acetic acid for another 30 min consecutively, and eluted mAbs were mixed. After buffer-exchanged to PBS, the eluted fractions were purified in the same way a second time with a regenerated SNA column to increase enrichment, and the fractions defined as SNA-bound fractions after buffer-exchanged to PBS. The flow-through fraction was applied to the regenerated SNA column, collected after incubation for 1 h to decrease specific binding fractions, and defined as SNA-non-bound fractions. SNA column regeneration was conducted by washing with 20 ml of 0.5 M lactose in PBS for 30 min, with 20 ml of 0.5 M lactose in 0.2 M acetic acid for another 30 min, followed by balancing with 30 ml PBS.

### Western Blot detection of Neu5Gc on mAbs

For Western Blot detection, each reduced sample (100 ng per lane) was separated by 4~20% gradient SDS-PAGE, and silver stained or blotted onto nitrocellulose membranes. Blotted membranes were blocked with TBST containing 1% human albumin overnight at 4 °C and subsequently incubated with affinity-purified chicken anti-Neu5Gc IgY for 4 h at room temperature (1:4,000 in TBST). Binding of the chicken anti-Neu5Gc IgY was detected using an HRP conjugated donkey anti-chicken IgY antibody for 1 h (1:10,000 in TBST), followed by incubation with SuperSignal West Pico Substrate (Pierce) as manufacturer’s recommendation, exposed to X-ray film and the film developed.

### Biotinylation of mAbs

Biotinylation of mAbs was performed according to the manufacturer’s instruction (Thermo Scientific). Briefly, each of the samples referred to in the paper was buffer-exchanged into 10 mM PBS (pH 7.4) with illustra NAP-5 column, and about 27 nmol Sulfo-NHS-Biotin was added into 200 μg of each sample with a final volume of 100 μl (molar fold of biotin reagent per protein sample was about 20). After incubation on ice for two hours, the mixture was buffer exchanged into 10 mM PBS (pH 7.4) with illustra NAP-5 column to remove excess biotin reagent.

### Denaturing of mAbs

Each of the samples referred to in the paper was mixed with guanidine with a final concentration of guanidine as 6 M, and after incubation at 37 °C for 30 minutes, the mixture was buffer exchanged into 10 mM PBS (pH 7.4) with illustra NAP-5 column. The denatured mAbs were immediately used in the ELISA assay as described below.

### ELISA detection of Neu5Gc on mAbs

For indirect capture ELISA, 96-well plates were coated with 1 μg of streptavidin (Sigma) per well overnight at 4 °C, blocked with TBST for 2 h, and 100 ng of each undenatured or denatured biotinylated samples was captured on the streptavidin coated wells by incubating at room temperature for 1 h. The samples were then incubated with affinity-purified chicken anti-Neu5Gc IgY for 1 h (1:1000 in TBST). Binding of IgY was detected using HRP-conjugated donkey anti-chicken IgY antibody (1:10,000 in TBST), and development with TMB (tetramethylbenzidin) peroxidase substrate (KPL, USA) followed by addition of 1 M sulphuric acid to terminate the chromogenic reaction, with the absorbance being measured at 495 nm. For competitive ELISA, 100 ng Cetuximab was coated in wells overnight at 4 °C, and blocked with TBST for 2 h. Different samples with serial dilution referred to in the paper was incubated with 1:1000 diluted anti-Neu5Gc IgY for 1 h, and the mixture was added into the wells, and then performed as the indirect capture ELISA as described above.

### BIAcore analysis

Real-time biomolecular interaction analysis between different samples referred to in the paper and anti-Neu5Gc IgY was performed using a BiaCore T200 instrument (GE Healthcare). Anti-Neu5Gc IgY was covalently immobilized at pH 5.0 in 10 mM sodium acetate buffer on a CM5 Chip (Series S) using EDC/NHS amine coupling chemistry. Ethanolamine was used to block remaining active carboxyl groups after immobilization. Regeneration was performed by 30 s injection of 10 mM glycine-HCl, pH 2.0. Various samples were diluted to 0.75 mg/mL with HBS-EP + running buffer (10 mM HEPES, 0.15 M NaCl, 3 mM EDTA, 0.05% polysorbate 20, pH 7.4) and injected sequentially into the microchip, and the binding signaling was recorded.

### icIEF analysis

icIEF analysis was performed using an iCE280 Analyzer (Convergent Bioscience) with a whole column imaging detection system. Samples were prepared at 0.3 mg/mL in the matrix containing 0.35% methylcellulose, 3% carrier ampholyte of pH 8-10.5 and 1% of pH 3-10 (GE Healthcare). Two pI markers of 9.77 and 8.46 (ProteinSimple) flanking the mAb being analyzed were used as internal standards for pI calibration of the charged species. The samples were focused and separated under the conditions of pre-focusing for 1 min under 1500 V, followed by focusing at 3000 V for another 10 min.

### IEC-HPLC analysis

Ion exchange chromatography-high performance liquid chromatography (IEC-HPLC) separation was performed using a weak cation exchange resin (ProPac WCX-10, 2.0 × 250 mm, 10 μm particle size, Dionex). Mobile Phase A (20 mM Sodium Phosphate, pH 7.10) and Mobile Phase B (200 mM Sodium Chloride in 20 mM Sodium Phosphate, pH 7.10) were used at a gradient of 0 to 20% B at a flow rate of 0.5 mL/minute to elute the samples with the column temperature of 50 °C and UV detection wavelength of 214 nm.

### Glycan mapping analysis

Glycans were released and labeled using GlycoWorks RapiFluor-MS N-Glycan Kit (Waters) according to the manufacture’s protocol^51^. Briefly, 15 μg of each mAb referred to in the paper was denatured by being diluted into a 28.8 μL solution of 1% (w/v) RG surfactant, and heated to approximately 95 °C over 2 min. Deglycosylation was completed by mixing the denatured solution with 1.2 μL of PNGase F and incubated at 50 °C for 5 min. The glycans were labeled by adding 12 μL of the RapiFluor-MS solution (addition of 335 μL of anhydrous DMF directly to one vial of 23 mg of RapiFluor-MS™ Reagent) to the deglycosylation solution and incubating at room temperature for 5 min. Labeled glycans were thereafter efficiently extracted from the reaction mixture using a GlycoWorks HILIC μElution plate. The labeled glycans were subsequently separated on a HILIC column (2.1 × 150 mm, BEH amide, 1.7 μm) at 60 °C and detected by an ACQUITY UPLC (Waters) fluorescence detector. Separations were performed at a 45-min gradient (72–55% B) and 0.4 ml/min flow rate using 30 mM ammonium formate, pH 4.7 as mobile phase A and 100% acetonitrile as mobile phase B. The labeled glycans were also characterized using sequential ESI-Q-TOF(Xevo G2, Waters) analysis with MS and positive ion mode to obtain mass profiling and structure elucidation. Resolution mode was used with a capillary potential of 3 kV, primary cone potential of 30 V, secondary cone potential of 4 V, desolvation gas temperature of 400 °C and flow rate of 600 ml/min. The molecular weight of each individual glycan from MS data and the corresponding fragment information from MS/MS data were input into GlycoWorkbench software to assign the glycoform, based on the composition of the glycan and the known glycosylation pathways for murine cells.

### Neu5Gc distribution analysis in intact Fc of mAbs with mass spectrometry

Each of the mAbs referred to in the paper with the final concentration of 1 mg/mL in the digestion buffer of 0.1 M TRIS, 4 mM EDTA, 1 mM cysteine, pH 7.4 containing 0.01 mg/mL papain (Roche) was incubated at 37 °C for 3 h, and the solution was then buffer-exchanged into 50 mM ammonium bicarbonate, pH 8.0, with illustra NAP-5 column, and then 1% of weight ratio of CPB was incubated with the samples at 37 °C for 30min to remove the terminal lysines. The samples were separated with a BEH C4 reversed phase column (1.7 μm, 2.1 mm × 15 cm) coupled with ESI-Q-TOF(Xevo G2, Waters) analysis. Separations were performed at 18-min gradient (20–35% B) and 0.4 ml/min flow rate using 0.1% formic acid as mobile phase A and acetonitrile containing 0.1% formic acid as mobile phase B. Capillary potential of 3 kV, primary cone potential of 35 V, secondary cone potential of 4 V, desolvation gas temperature of 400 °C and flow rate of 600 ml/min with positive mode were used in the mass spectrometry analysis. The Fc peaks in the mass spectrogram were deconvoluted using Biopharmalynx software (Waters) with input of primary sequence of each mAb Fc and glycoforms (Supplementary Fig. 4) resulted from the above analysis, and the deconvoluted peaks were automatically assigned by the software followed by manual calibration one by one in conformance with the combination of two of the above-mentioned known glycoforms.

## Additional Information

**How to cite this article**: Yu, C. *et al.* At least two Fc Neu5Gc residues of monoclonal antibodies are required for binding to anti-Neu5Gc antibody. *Sci. Rep.* **6**, 20029; doi: 10.1038/srep20029 (2016).

## Supplementary Material

Supplementary Information
