# Erratum: At least two Fc Neu5Gc residues of monoclonal antibodies are required for binding to anti-Neu5Gc antibody

**DOI:** 10.1038/srep46385

**Published:** 2017-06-30

**Authors:** Chuanfei Yu, Kai Gao, Lei Zhu, Wenbo Wang, Lan Wang, Feng Zhang, Chunyu Liu, Meng Li, Mark R. Wormald, Pauline M. Rudd, Junzhi Wang

Scientific Reports 6: Article number: 20029; 10.1038/srep20029 published online: 01 29 2016; updated: 06 30 2017.

The original HTML version of this Article listed an incorrect volume number. This has now been corrected in the HTML version; the PDF version was correct at the time of publication.

