# Supplementary material for: At least two Fc Neu5Gc residues of monoclonal antibodies are required for binding to anti-Neu5Gc antibody
Source: Sci Rep. 2016 Jan 29;7:20029. doi: 10.1038/srep20029 (PMC4731815; doi:10.1038/srep20029)
Supplement: Supplementary Information [file srep20029-s1.doc]

**Supplementary Information for *SCIENTIFIC REPORTS Articles***

**At least two Fc Neu5Gc residues of monoclonal antibodies are required for binding to anti-Neu5Gc antibody**

Chuanfei Yu, Kai Gao, Lei Zhu, Wenbo Wang, Feng Zhang, Lan Wang, Chunyu Liu, Meng Li, Mark R Wormald, Pauline M. Rudd, Junzhi Wang

**Supplementary Table 1. Sialic acids on various mAbs expressed in CHO, SP2/0 and NS0 cells.**

|  | Antibody | Total sialic acid  (mol/mol antibody) | %Neu5Gc | Neu5Gc content  (mol/mol antibody) |
| --- | --- | --- | --- | --- |
| Expressed in CHO cells | Bevacizumab | 0.022 | 2.43 | <0.001 |
| Expressed in SP2/0 cells | ATI355 | 0.106 | 99.31 | 0.105 |
|  | Canakinumab | 0.009 | 83.60 | 0.008 |
|  | Ustekinumab | 0.548 | 99.21 | 0.544 |
|  | Epratuzumab | 0.046 | 77.39 | 0.035 |
|  | Golimumab | 0.328 | 98.76 | 0.324 |
|  | Cetuximab | 1.734 | 97.42 | 1.689 |
|  | Basiliximab | 0.007 | 96.63 | 0.007 |
|  | Infliximab | 0.088 | 96.82 | 0.085 |
| Expressed in NS0 cells | Ramucirumab | 0.154 | 97.88 | 0.151 |
|  | Belimumab | 0.030 | 97.12 | 0.029 |
|  | Palivizumab | 0.024 | 95.53 | 0.023 |
|  | Sirukumab | 0.062 | 96.24 | 0.060 |
|  | Dalotuzumab | 0.043 | 98.09 | 0.043 |
|  | Ofatumumab | 0.085 | 95.93 | 0.082 |
|  | Nimotuzumab | 0.091 | 93.94 | 0.085 |
|  | Daclizumab | 0.029 | 93.59 | 0.027 |
| SNA-bound Ustekinumab | | 2.327 | 85.63 | 1.993 |
| SNA-bound Ramucirumab | | 2.008 | 83.76 | 1.682 |

Sialic acids were released by mild acid, derivatized with 1,2-diamino-4,5-methyl dioxybenzene (DMB), and analyzed by HPLC as described in Methods.


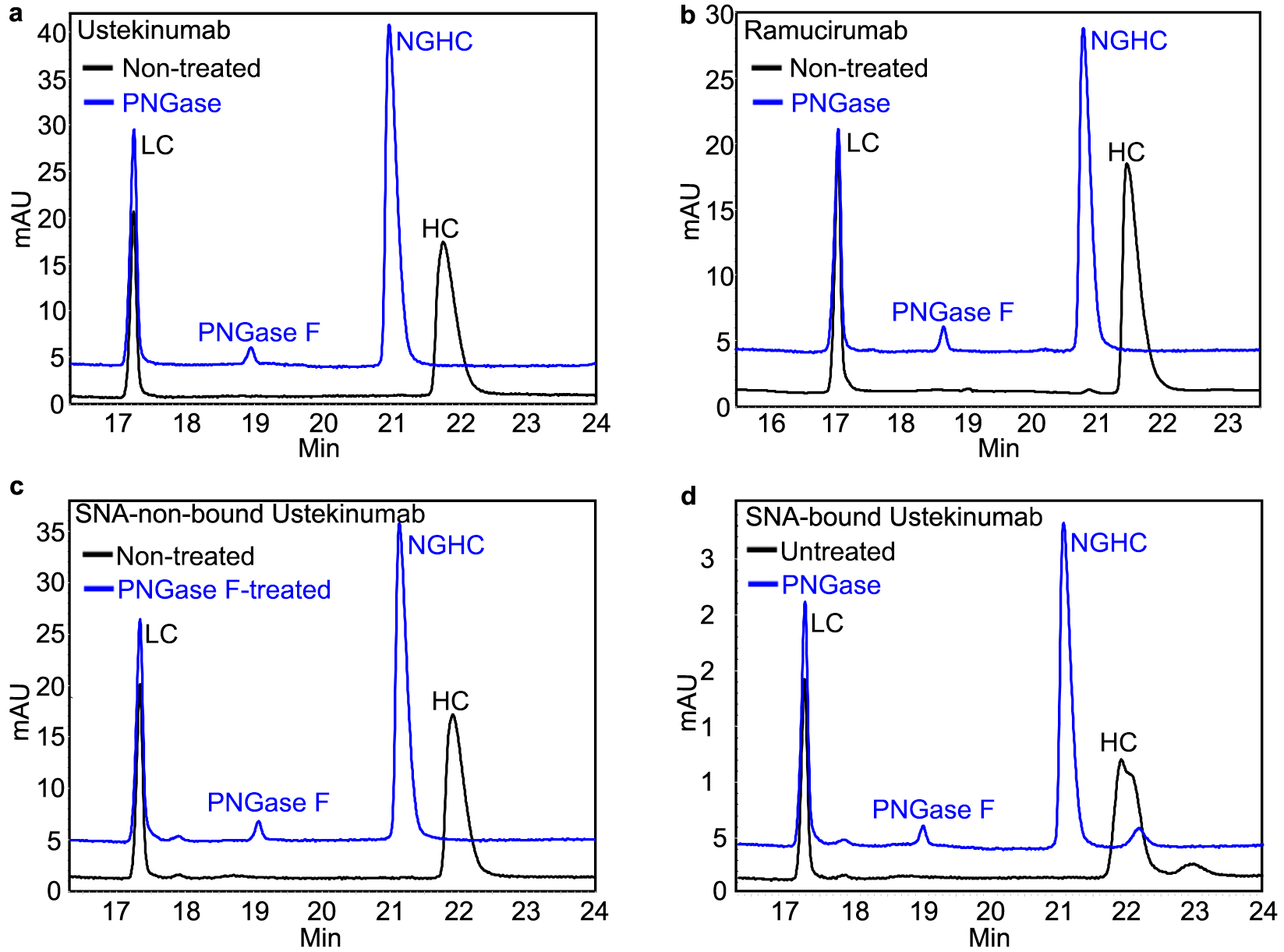


**Supplementary Figure 1. CE-SDS analysis of the antibodies to evaluate the N-glycan removal efficiency.**

N-glycans were removed from Ustekinumab (a), Ramucirumab (b), SNA-non-bound Ustekinumab (c) and SNA-bound Ustekinumab (d) by PNGase F, and the samples were separated by CE-SDS. The peaks of HC (Heavy Chain), LC (Light Chain), NGHC (NonGlycosylated Heavy Chain) and PNGase F are indicated.


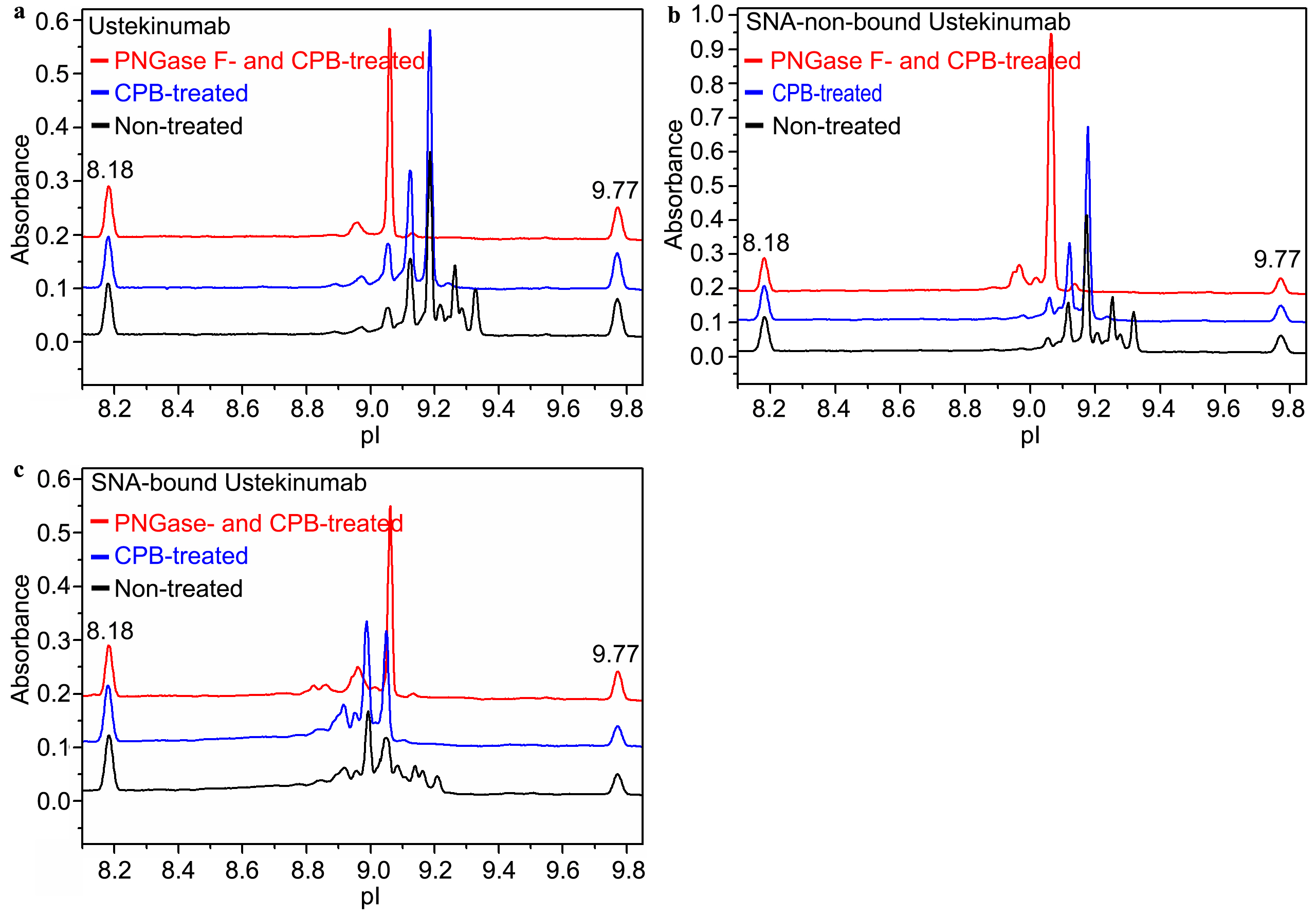


**Supplementary Figure 2. IcIEF analysis to evaluate the charge heterogeneity of the antibodies.**

Ustekinumab (a), SNA-non-bound Ustekinumab (b) and SNA-bound Ustekinumab (c) were trimmed of c-terminal lysine by CPB, indicating the removal of the c-terminal lysine induced basic variants. N-glycans were subsequently removed by PNGase F, indicating the elimination of N-glycan terminal sialic acid/s induced acidic variants as shown by icIEF analysis. The peaks of the samples were flanked by two PI markers (8.18 and 9.77).


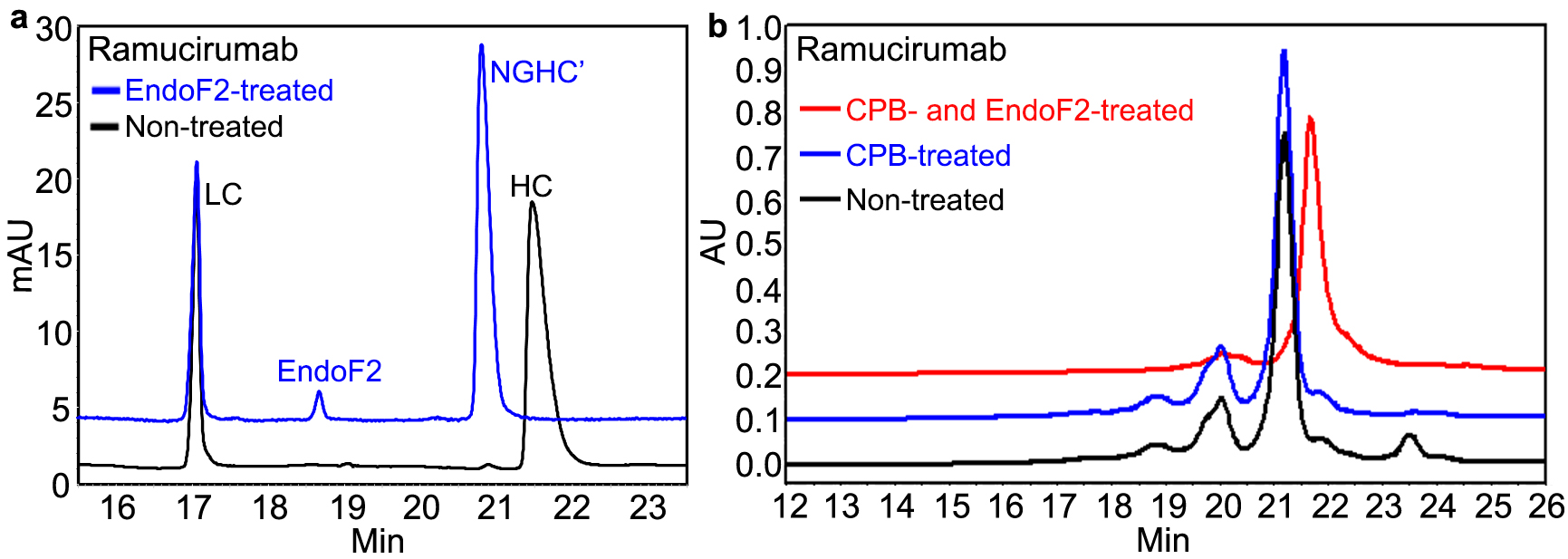


**Supplementary Figure 3. IEC-HPLC analysis to evaluate the charge heterogeneity of the antibodies.**

(a) N-glycans were removed from Ramucirumab by EndoF2, and the samples separated by CE-SDS. The peaks of HC (Heavy Chain), LC (Light Chain), NGHC’ (Non-Glycosylated Heavy Chain’) and EndoF2 are indicated. EndoF2 cleaves the glucosidic bond between the first and second N-acetyl glucosamine residues at the non-reducing end of the N-glycan, leaving an N-acetyl glucosamine residue attached to the asparagine of the antibody. This was named the EndoF2-treated HC (NGHC’) to differentiate it from PNGase F-treated HC (NGHC). (b) C-terminal lysine induced basic variants of Ramucirumab were eliminated by CPB treatment, and subsequently the N-glycan terminal sialic acid induced acidic variants were removed by EndoF2 treatment, as shown by IEC-HPLC analysis.


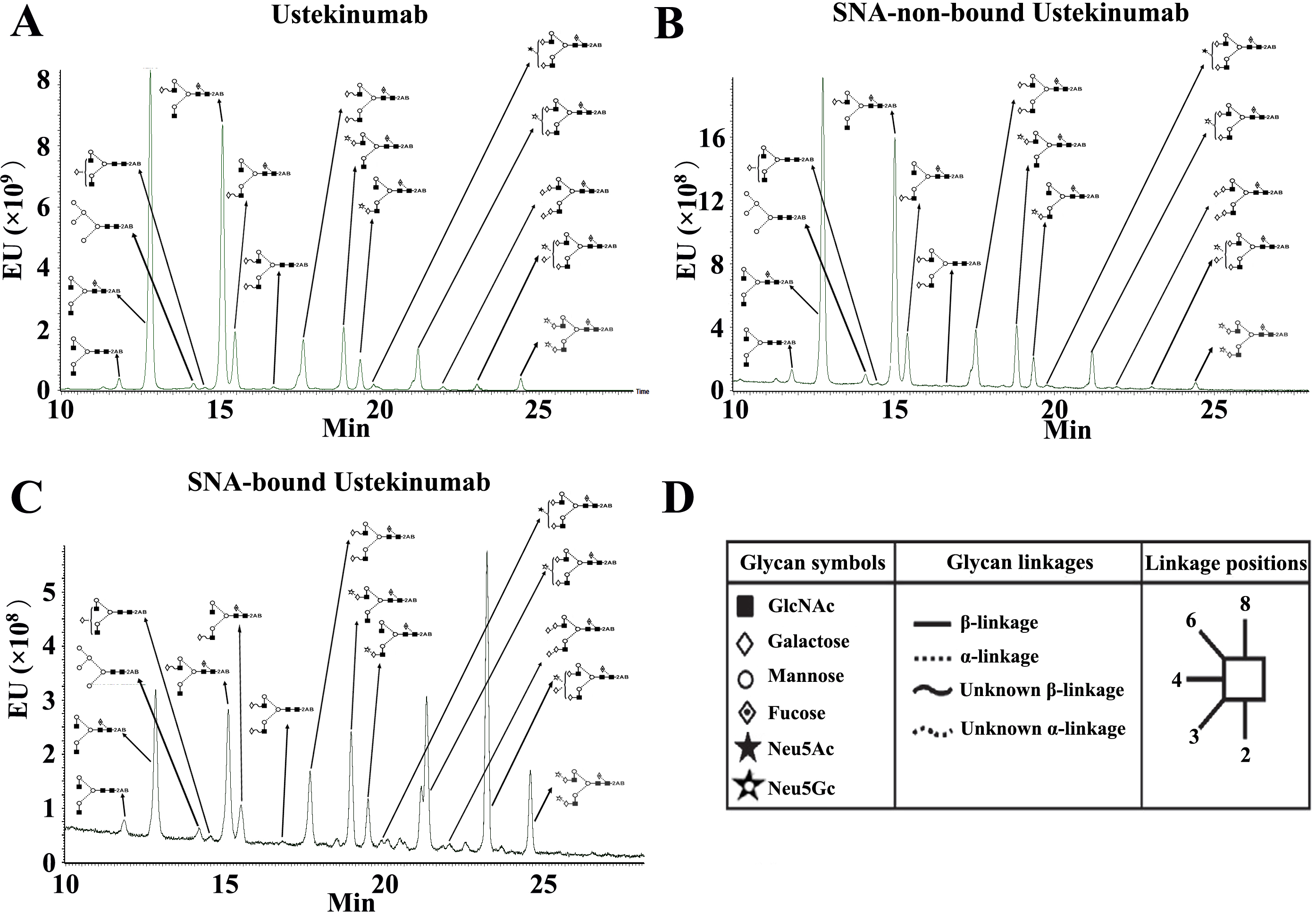


**Supplementary Figure 4. N-glycan profile analysis of Ustekinumab, SNA-non-bound and -bound Ustekinumab.**

N-glycans were released from Ustekinumab (a), SNA-non-bound Ustekinumab (b) and SNA- bound Ustekinumab (c) with PNGase F, and labeled with fluorescent RapiFluor-MS™ Reagent. The glycan pool was separated by HILIC-UPLC with quantification, and coupled with MS detection for confirmation of the structural assignments (data not shown) based on the assignments from the composition and the known glycosylation pathways for murine cells. The structures were also orthogonally confirmed by glucose unit ladders and the corresponding Glyobase database developed by the National Institute for Bioprocessing Research and Training in Ireland (data not shown). The glycan profiles corresponding to each peak were indicated. (d) N-Glycan nomenclature with Oxford notation is indicated.


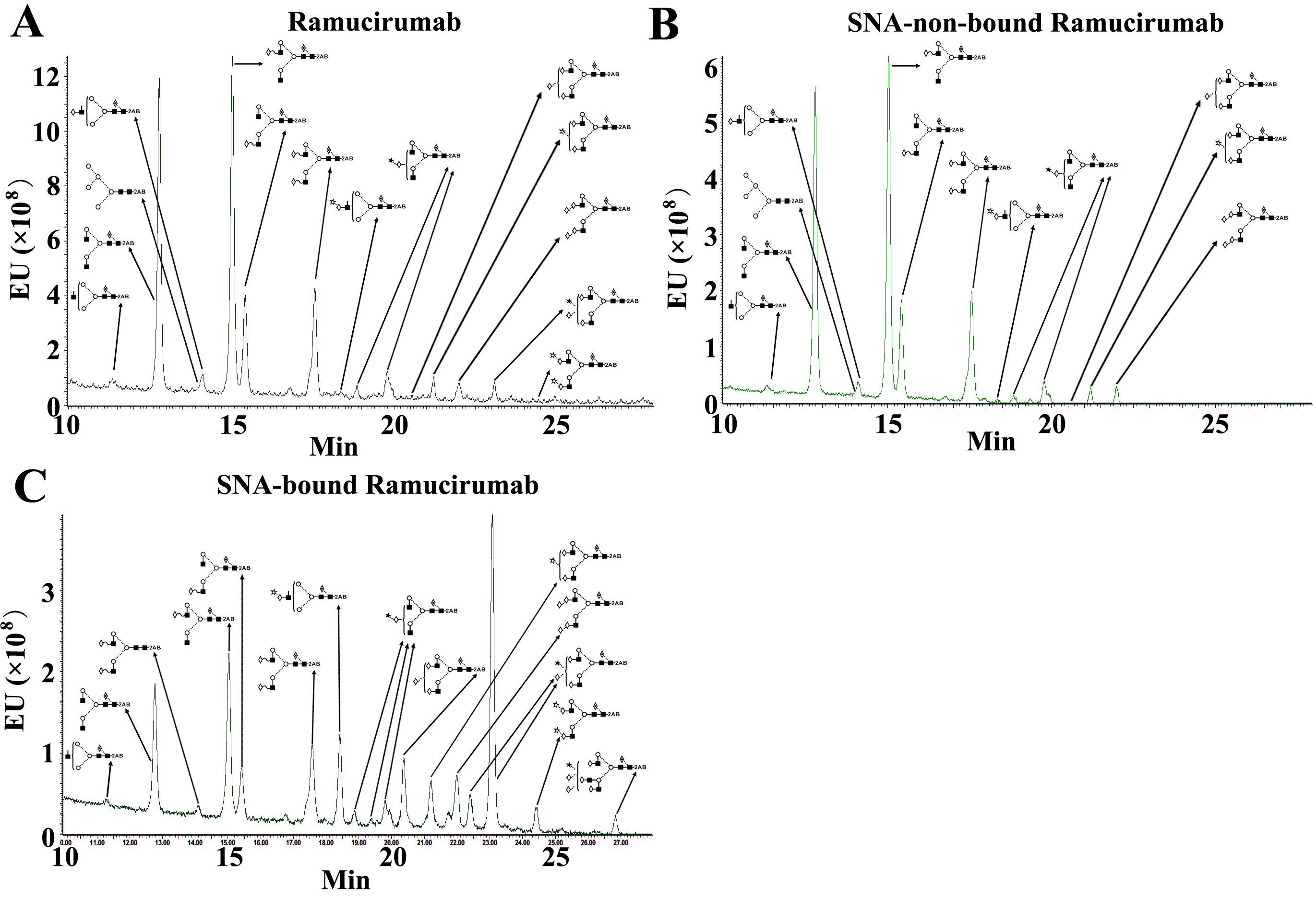


**Supplementary Figure 5. N-glycan profile from the LC analysis of Ramucirumab, SNA-non-bound and -bound Ramucirumab.**

N-glycans were released from Ramucirumab (a), SNA-non-bound Ramucirumab (b) and SNA-bound Ramucirumab (c) with PNGase F, and labeled with fluorescent RapiFluor-MS™ Reagent. The glycan pool was separated by HILIC-UPLC with quantification, and coupled with MS detection for confirmation of the structural assignments (data not shown) based on the assignments of the composition and the known glycosylation pathways for murine cells. The monosaccharide sequence and linkages were orthogonally determined by glucose unit ladders and the corresponding Glycobase database developed by the National Institute for Bioprocessing Research and Training in Ireland (data not shown). The glycan structures corresponding to each peak are indicated.
